# One-step fabrication of carbonaceous adsorbent from corncob for enhancing adsorption capability of methylene blue removal

**DOI:** 10.1038/s41598-020-68591-1

**Published:** 2020-07-27

**Authors:** Youming Wang, Yulong Zhou, Guojing Jiang, Peirong Chen, Zhen Chen

**Affiliations:** 1 Department of Applied Chemistry, School of Natural Science, Anhui Agricultural University, Hefei, 230036 Anhui China; 2 Key Laboratory of Biomass and Energy of Education, Department of Anhui Province, Anhui Agricultural University, Hefei, 230036 China

**Keywords:** Bioinspired materials, Environmental monitoring

## Abstract

A novel and simple method was described for preparation of carbonaceous adsorbent (CA) from corncob under phosphoric acid conditions. The method succeeded to introduce oxygen-containing groups onto the product surface through hydrothermal carbonization (HTC) at low temperature of 160 °C. Adsorption of methylene blue (MB) was studied systematically through the effect of pH, contact time and initial dye concentrations. The MB adsorption kinetics and isotherms experiments showed that Langmuir model and pseudo-second-order model could better describe the adsorption behavior, with a maximum adsorption capacity of MB was 140.25 mg/g. The high adsorption capacity could be ascribed to the presence of surface oxygen-containing functional groups and pore channels. In conclusion, it could be a potential adsorbent in the removal of methylene blue from wastewater.

## Introduction

The presence of dyes in the wastewater can cause various detriment effects on human health and living organisms due to their not biodegradable and become a major environmental concern problem. These dyes are introduced in the freshwater from many industries, including cosmetics, leather, textile, paper, printing and dyeing industries^1–8^. Methylene blue (MB) is one of the most typical dye pollutants in aqueous systems^9^. Until now, various techniques such as photochemical oxidation, reverse osmosis, membrane filtration, adsorption and ozonation have been developed for the purification of organic contaminants from aqueous solutions^10–18^. Among these techniques, adsorption is regarded as a superior technique for treating wastewater considering its process simplicity, nontoxic and inexpensive. In particular, tremendous attention has been attracted to the study of different types of biomass-derived adsorbents removing organic pollutants in waterways^19–25^.

According to a recent China’s National Bureau of Statistics report, the Chinese annual yield of corn in 2017 was more than 21.5 million tons. The radio between corn gain and corncob may be about 100:18^26^, rough estimate 3.8 million tons of corncobs could be generated in China every year. Corncob is a sort of environment friendly and renewable resource, widely existing in biomass. Currently, most of the corncobs are burned as low-grade fuels and not fully utilized, which cause both environmental pollution and natural resources waste. Therefore, reasonable and harmless utilization of corncob is important to change waste into valuables.

However, to date, the most widely biomass-derived adsorbents were prepared under the most demanding conditions with high pressure and high temperature^27–29^. In addition, widespread application is seriously restricted because of the relatively poor capability^30^. Therefore, chemical modification method is widely used presently to enhance their capabilities of biochar by introduction of oxygen-containing groups^31–35^. These oxygen-containing groups on the surface serve as adsorption sites for organic pollutants in solution. For example, Xu et al. reported that citric acid-promoted water hyacinth (*Eichornia crassipes*) was an efficient method for enhancing the adsorption capability of biochar for MB^36^. Rhamnolipid was employed to enhance the adsorption capability of biochar for removal of MB^37^. However, there have been few reports that phosphorylated modification at low temperature is investigated for improving adsorption capability of biochar in wastewater treatment.

As one of most frequently used agents for chemical modifications, phosphoric acid is preferred recently for environmental and production costs. The phosphoric acid has played a vital role in the HTC process including the degradation of biomass and the formation of oxygen-containing surface functional groups. Thus, the main purpose of this research was to develop an effective modification method for preparing an alternative corncob-derived adsorbent with H_3_PO_4_ at low temperature. These works help not only to improve adsorption capability of biochar but also greatly reduce the preparation temperature. In addition, we aim to characterize the properties of as-prepared adsorbent and study their sorption behaviors for MB.

## Methods

### Materials

Corncob used in this study was obtained from the a farm around Fuyang City, Anhui Province of China. Before processing, the corncob was washed by water, dried at 105 °C for 24 h and smashed to pass through a 0.15 mm (100 mesh) sieve. Methylene blue (MB) was purchased from Sinopharm in Beijing, All chemicals were of analytical grades from the Shantou Xilong Chemical Factory, China.

### Preparation of the corncob-derived adsorbent

The corncob powder (5 g) was added to 40 mL distilled water with a certain amount of phosphoric acid (5 mL) in a 100 mL Telon-lined reactor, then sonicated for 1 h. The resulting mixture was heated to desired temperature for 10 h in the oven. After cooling, the sample was washed with distilled water, and then obtained by drying at 85 °C over night.

### Characterization techniques

The microstructure and functional groups of the samples was studied by the X-ray diffraction (XRD), Brunauer–Emmet–Teller (BET) and infrared spectroscopy (IR). Analyses of the morphology of the sample were conducted by a scanning electron microscope (SEM), Hitachi S-4800. The MB concentration was analyzed by using a UV–Vis spectrophotometer.

### Adsorption experiments

All adsorption experiments were carried out in 50 mL polypropylene (PP) centrifuge tubes. With a procedure, the effect of initial pH, contact time and initial MB concentrations on the adsorption of MB were examined in a series of experiments. Analytical simples were taken from the mixture solution at predetermined time intervals during the adsorption. The content of MB in the PP centrifuge tubes was determined by means of UV spectrophotometer.

For tests on the adsorption isotherm, the adsorbent concentration of 1 g/L and the MB concentration varied from 5 to 300 mg/L under constant shaking on a rotary shaker at room temperature for 8 h.

### Desorption studies

In the experiments, adding a certain amount of the MB-adsorbed CA powder to 50 mL of 0.1 M HCl solution and stirring continuously for 120 min at ambient temperature, and then the MB-desorbed CA powders were filtered from the solution. After regeneration, the regenerated adsorbents were recycled 4 times in the recycle adsorption study. The experiments were performed at the same initial conditions.

## Results and discussion

### Characterization of adsorbent

**Figure 1:**
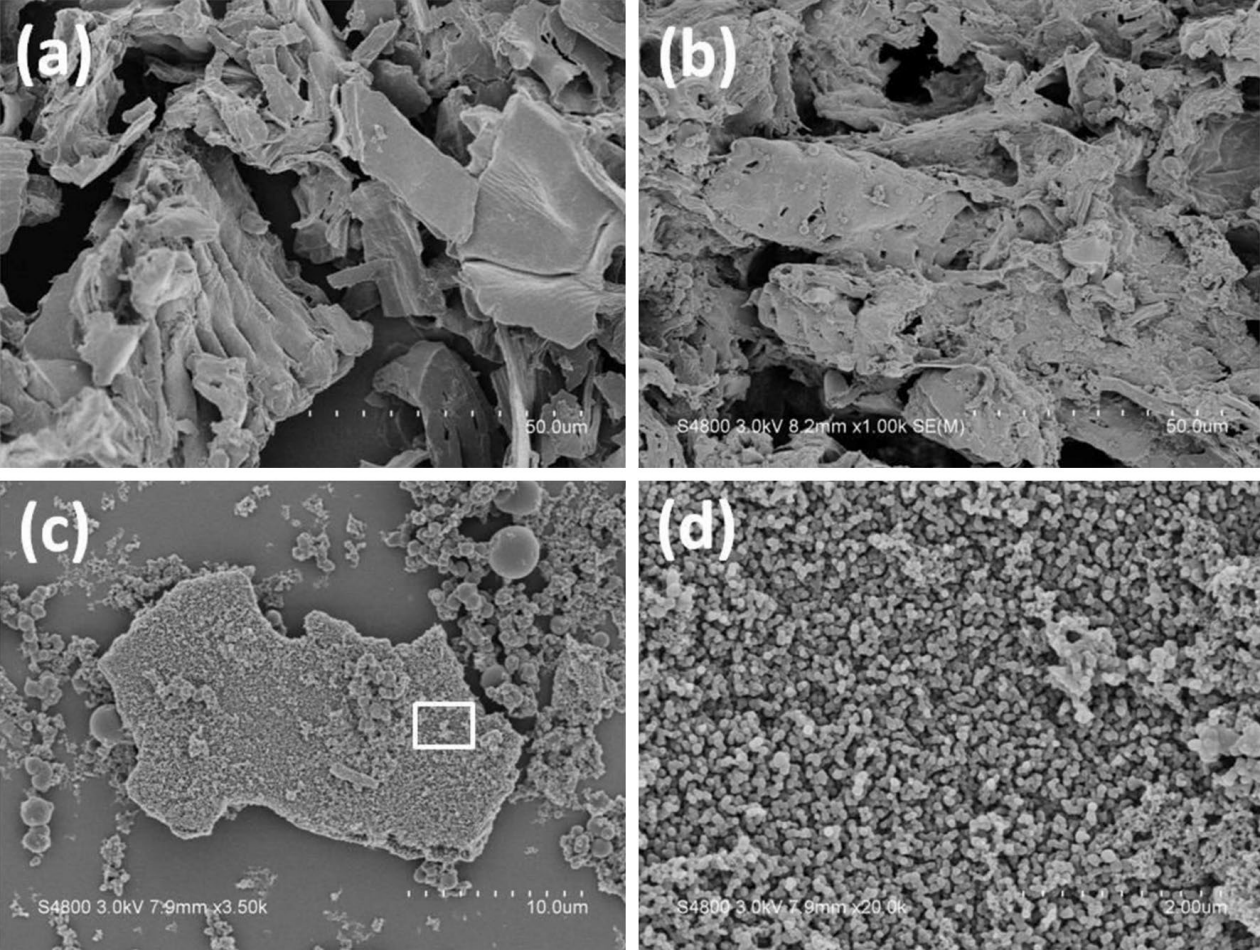
SEM images of corncob powder (**a**) before and (**b**) after hydrothermal carbonization at 160 °C for 10 h without adding H_3_PO_4_, (**c**) after hydrothermal carbonization at 160 °C for 10 h adding H_3_PO_4_ and the enlarged view of the black frame of **c** (**d**).

In order to highlight the effect of H_3_PO_4_ in HTC process, the carbonized corncob prepared without adding H_3_PO_4_ was employed as a reference. The SEM images showed that the surface of corncob relative smooth with some flakes (Fig. 1a,b).

**Figure 2:**
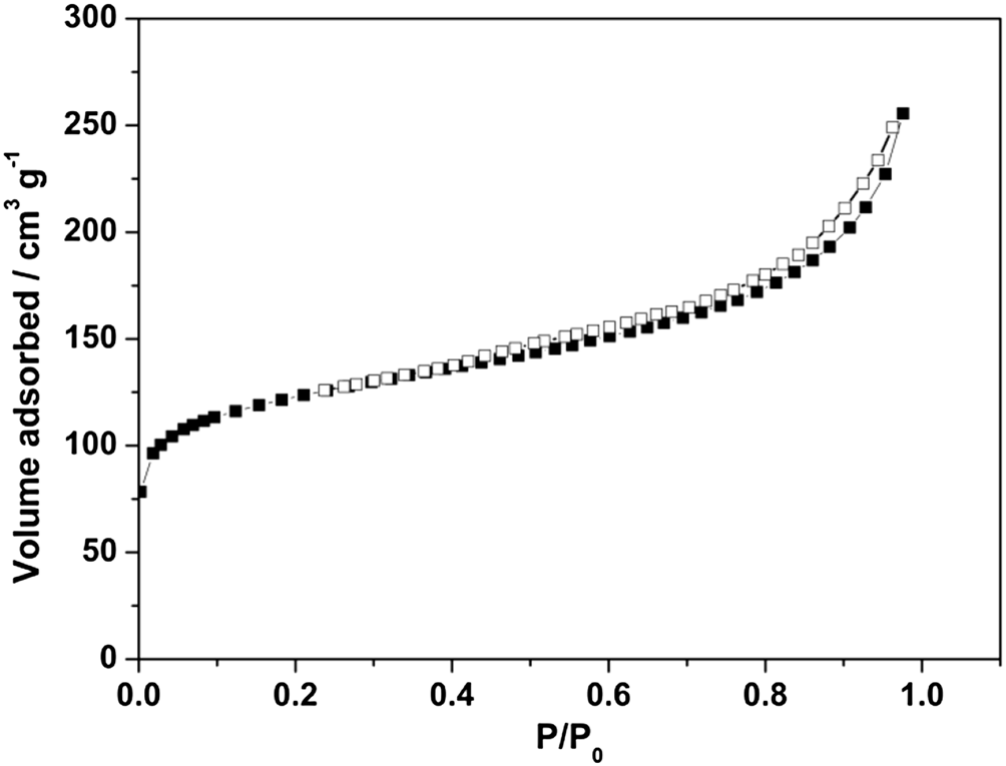
Nitrogen adsorption–desorption isotherms of carbonaceous adsorbent (CA).

After hydrothermal reaction in the presence of phosphoric acid, the SEM images showed that the formation of a great deal of particles and aggregated dispersion on the surface (Fig. 1c). Detailed information on the structure was further obtained by enlarging SEM images. Figure 1d illustrated that the resulting particles are regularly distributed. These results revealed that adding H_3_PO_4_ could successfully increase the surface area, resulting in the increase of pore volume. Nitrogen adsorption/desorption isotherms in Fig. 2 were used to analyze structurally pores of the carbon samples. CA showed the surface area of 480 m^2^/g.

**Figure 3:**
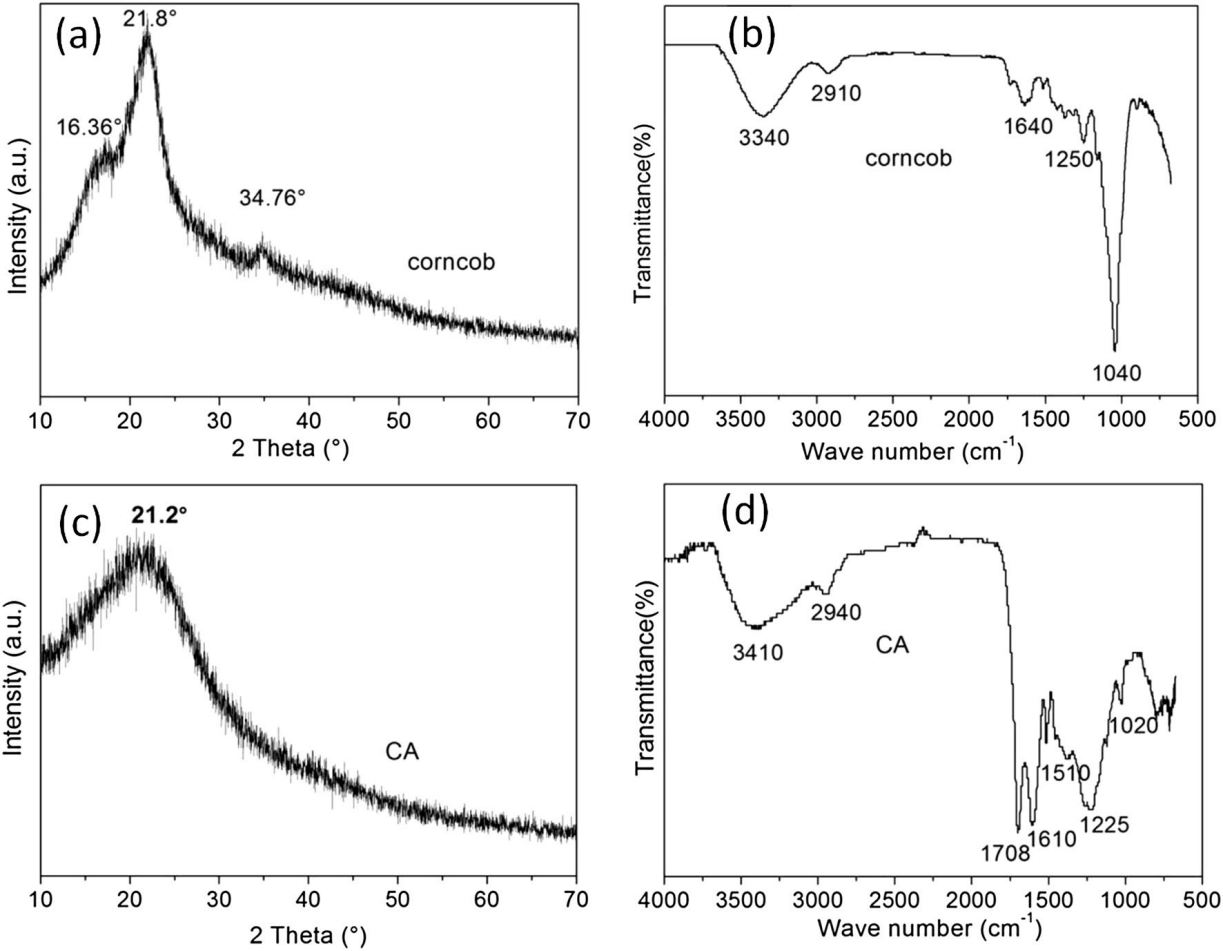
XRD pattern of corncob powder (**a**) and CA (**c**), FTIR spectra of corncob powder (**b**) and CA (**d**).

The phases of the raw material and hydrothermal carbonized samples were observed by the XRD patterns. In comparison with the raw corncob, the characteristic peaks of the prepared CA became more obvious, indicating that single HTC process only depleted impurities but without destroying the “core’’ structure of corncob. As shown in Fig. 3c, the XRD pattern of the prepared CA exhibited one broad diffraction peaks (2*θ* = 15–25°), indicating that the prepared CA existed in an amorphous carbon composed of aromatic carbon sheets form^38^. This result clearly suggests that the presence of phosphoric acid yields a carbonaceous structure composed of aromatic carbon sheets under HTC condition.

The adsorption efficiency of CA depends on the number and chemical reactivity of surface functional groups^39^. The FT-IR technique was used to identify the characteristic functional groups. According to Fig. 3d, the FT-IR spectra of CA was obvious difference from that of the raw corncob, which imply that major spectral changes occurred in the presence of phosphoric acid after HTC process. The bands at 1,040 assigned to aromatic C–O have nearly disappeared, while C=C peak at 1,610 cm^−1^ and the C=O peak at 1708 cm^−1^ were enhanced. All of these observations were in agreement with the structural characteristics of standard HTC process^40^, indicating that the presence of phosphoric acid promoted the carbonization of lignocellulosic biomass through HTC process at low temperature (160 °C) and introduced carboxyl group into the structure of the raw corncob^41^. These oxygen-containing functional groups can play a significant role in enhancing CA adsorption efficiency by means of specific adsorption such as H-bonding and π–π interaction^42^.

### Adsorption of MB

#### Effect of the initial pH

**Figure 4:**
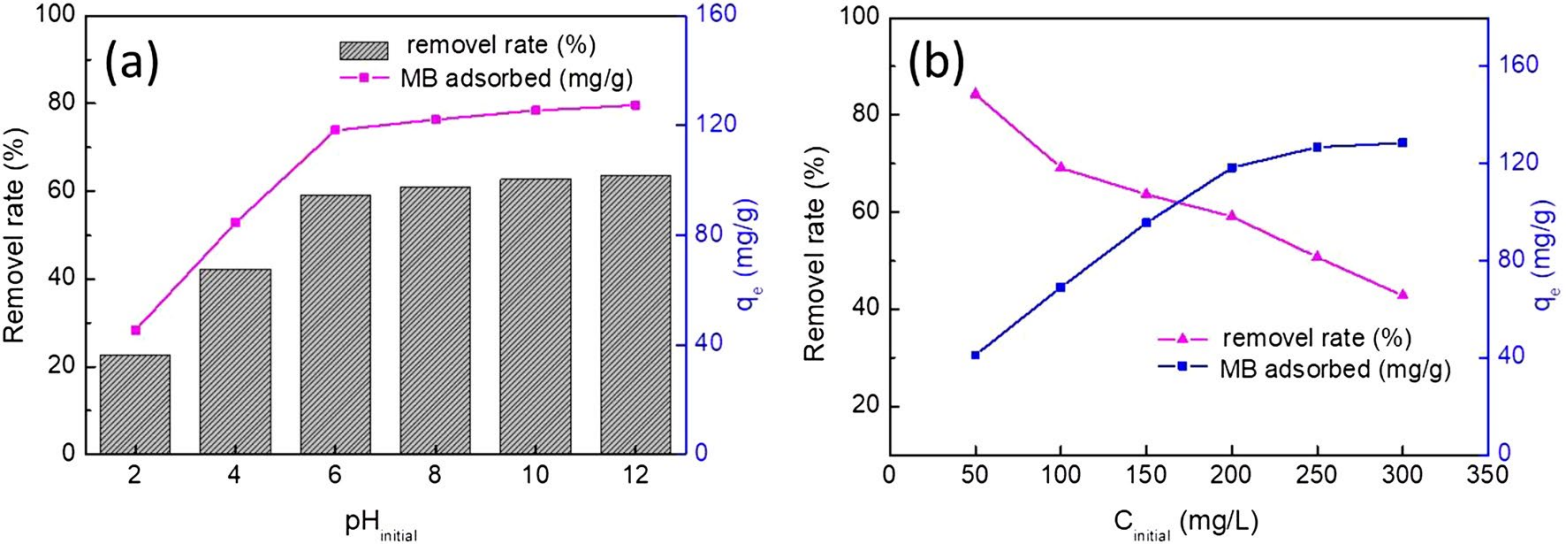
Effect of (**a**) pH and initial concentrations (**b**) for MB adsorbed on CA.

The pH is one of the most important parameter in the adsorption process, which regulates the adsorbents surface charge and influences the particles adsorption affinity in various aqueous solutions^43^. In order to evaluate the influence of pH on adsorption of MB, experiments were performed under the following condition: adsorbent dose 1.0 g/L, initial MB concentration 200 mg/L, pH (from 2 to 12), reaction temperature 20 ± 1 ℃ and contact time 120 min. The removal ratio of MB increased with an increase of pH from 2 to 7 and thereafter slightly increased with increasing pH range from 7 to 12, and the maximum removal was observed at pH 7 (Fig. 4a). At very low pH, lower adsorption efficiency was recorded due to the H^+^ competed with MB molecules for the negative active sites on the adsorbent surface. Thus cationic dye (MB) cannot move toward the positively charge surface area of adsorbent as a result of electrostatic repulsive force and hence a lower removal was observed at low pH values^44^. At high pH, a significant enhancement in adsorption was observed owing to electrostatic attraction increased between the adsorbent and cationic dye (MB)^26,45^. Therefore, all further adsorption experiments were performed at pH values of 7.

#### Effect of the initial MB concentrations

Adsorption behavior of cationic dye onto the adsorbent at various MB concentrations of 50–300 mg/L was also investigated as shown in Fig. 4b. The figure showed that the amount of MB adsorption adsorbed increased with increasing in the initial MB concentration, but the removal efficiency decreased from 81.28 to 42.81%. The percentage of adsorption decreased may be explained that sorption sites on the surface area of adsorbent were insufficient to meet much more MB molecules available in aqueous solution^18,46^.

#### Effect of the contact time

**Figure 5:**
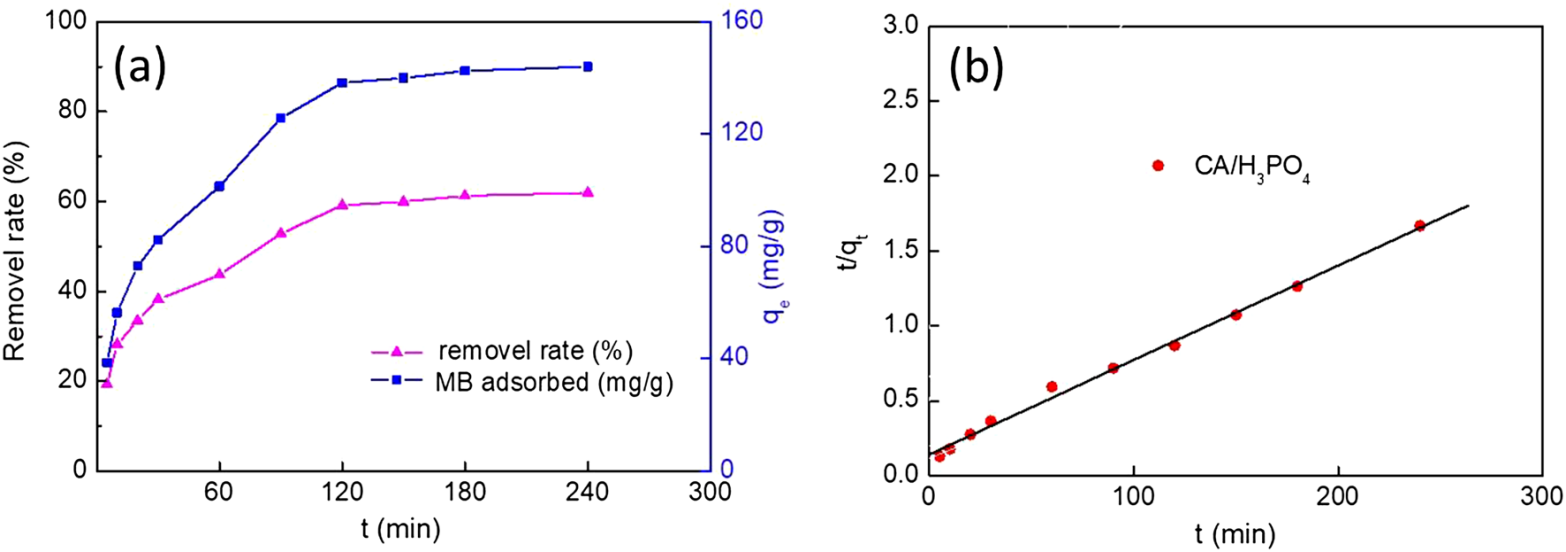
Effect of contact time on MB adsorbed on CA (**a**), and the pseudo second-order kinetic plots of MB adsorption on CA (**b**).

To evaluate the effect of contact time on the adsorption of MB molecules, the uptake capacity of the adsorbent with contact time was tested in Fig. 5a. It can be observed that removal efficiency was characterized by a rapid sorption phase followed by a slow phase as the contact time increases from 0 to 240 min. The results revealed that an initial very fast step where > 80% of MB molecules were adsorbed within the first 120 min and a slow phase where equilibrium was attained within the later 120 min, which might be attribute to that the availability of a large number of active sites on the surface of adsorbent at the initial stage were provided in aqueous solutions^2,47^.

### Adsorption isotherm

The adsorption isotherm studies are basic requirements for optimization of the adsorption systems, describing the relationship between MB molecules adsorbed onto the adsorbent and MB molecules in aqueous solution^48^. In current study, two types of well-known adsorption isotherms models Langmuir and Freundlich were fitted to analyze the adsorption equilibrium data^16^.

Langmuir isotherm model, which is valid for a monolayer adsorption with a homogeneous distribution of the adsorption sites and sorption energies, is mathematically given as follows^49^:

1 $$ \tfrac{{c_{e} }}{{q_{e} }} = \tfrac{1}{{K_{{\text{L}}} q_{m} }} + \tfrac{{c_{e} }}{{q_{m} }} $$

where the values of *q*_e_ (mg/g) and *c*_e_ (mg/L) represent the sorption capacity at equilibrium and the equilibrium concentration, respectively; *K*_L_ (L/mg) is the Langmuir isotherm equilibrium constant; *q*_m_ (mg/g) is the maximum sorption capacity.

Freundlich isotherm model is derived by assuming that the MB molecules can be applied for multilayer sorption. It is used to describe heterogeneous systems by the following equation^50^:

2 $$ \ln q_{e} = \ln K_{\text{f}} + \frac{1}{n}\ln c_{\text{e}} $$

where the values of *q*_e_ (mg/g) and *c*_e_ (mg/L) represent the sorption capacity at equilibrium and the equilibrium concentration, respectively; *K*_f_ (mg/g) is the Freundlich isotherm constant; indicating the adsorption capacity related to bond strength; 1/n is also Freundlich isotherm constant that stand for adsorption intensity.

**Figure 6:**
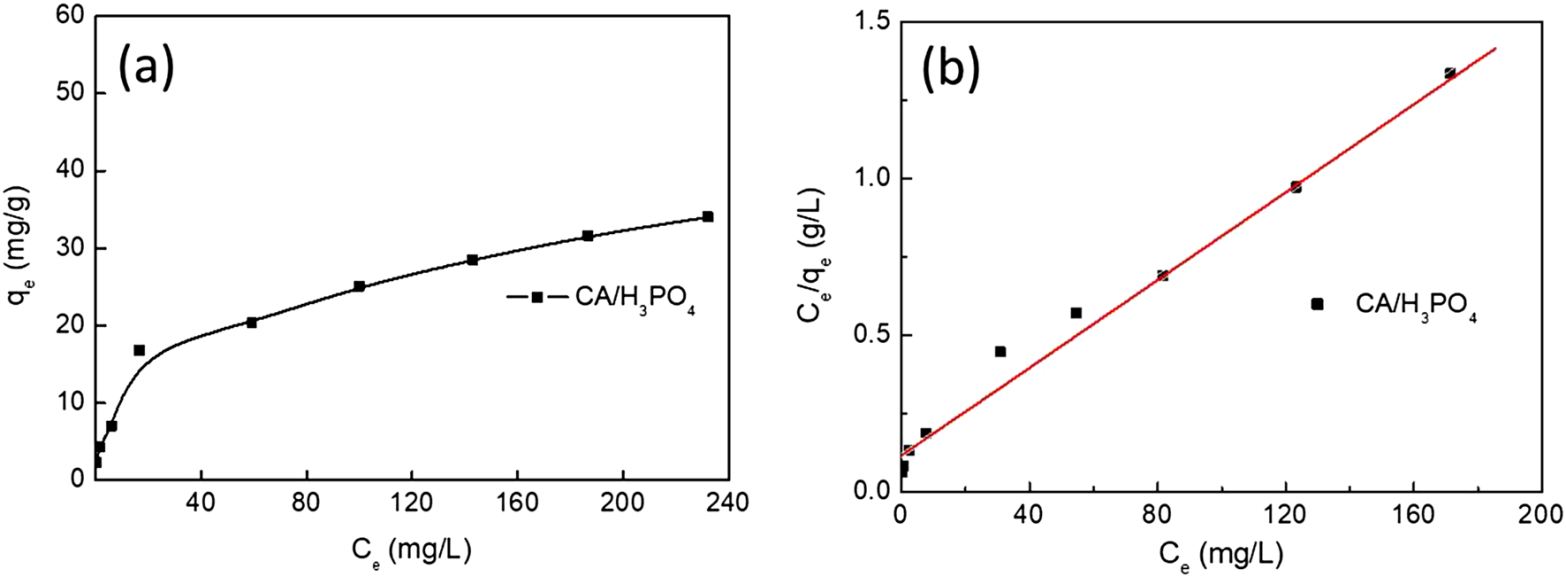
Adsorption isotherms for MB on CA (**a**), and the Langmuir isotherm plots for adsorption of MB on CA (**b**).

The adsorption isotherms of MB molecules onto the adsorbent were shown in Fig. 6. The Langmuir and Freundlich isotherms parameters of corncob and CA were given in Table 1. The results show that the value of *R*^2^ for Langmuir isotherm model (0.993 for CA) was higher than that of Freundlich isotherm model (0.938 for CA), indicating that Langmuir isotherm model was better fitted to describe the adsorption of MB molecules onto CA. In addition, the calculated maximum sorption capacity of MB by Langmuir model was 140.25 mg/g in Fig. 6a, which was quite close to their corresponding experimental data (128.43 mg/g). These results suggest that the monolayer adsorption occur on the surface of the CA for cationic dye^31^.

**Table 1:** Isotherms parameters of MB on CA.

Isothermal models	Parameters	CA
Langmuir	*K*_L_ (L/mg)	0.5795
	*q*_m_ (mg/g)	140.25
	*R*^2^	0.993
Freundlich	*K*_F_ (mg/g (1/mg)^1/n^)	87.102
	*n*	5.1524
	*R*^2^	0.938

### Adsorption kinetics

Adsorption kinetic study is important to provide the vital information about the mechanism of adsorption process and the reaction pathways in the treatment of waste water. Thus, to investigate the adsorption kinetics, three types of well-known kinetic models were studied: pseudo-first-order kinetic model, pseudo-second-order kinetic model and intra-particle diffusion model. The three models were expressed as follows^51^:

3 $$ {\text{Pseudo-first-order}}\;{\text{kinetic}}\;{\text{equation}}{:}\;\;\;\ln (q_{e} - q_{t} ) = \ln q_{e} - k_{1} t $$

4 $$ {\text{Pseudo-second-order}}\;\;{\text{kinetic}}\;\;{\text{equation}}{:}\;\tfrac{t}{{q_{t} }} = \tfrac{1}{{k_{2} q_{e}^{2} }} + \tfrac{t}{{q_{e} }} $$

5 $$ {\text{Intra-particle diffusion model}}{:}\;\;q_{t} = k_{i} t^{\frac{1}{2}} + C $$

where the values of *q*_e_ (mg/g) and *q*_t_ (mg/g) are the amounts of MB absorbed at equilibrium and at time *t* (min); *k*_1_ (1/min) and *k*_2_ (g/(mg min)) are the equilibrium rate constant of pseudo-first-order adsorption and the equilibrium rate constant of pseudo-second-order adsorption, respectively. *k*_i_ (mg/(g min^1/2^)) is the intra-particle diffusion rate constant; C (mg/g) is a constant.

Three types of kinetic models for MB molecules onto adsorbent were investigated. The calculated relevant parameters of the three kinetic equations for the adsorption of MB molecules onto adsorbent were shown in Table 2. Comparison of the correlation coefficient (*R*^2^) values for three models suggested that the pseudo-second-order model was better fitted to describe the adsorption process due to its highest values. However, the value of the correlation coefficient (*R*^2^ < 0.98) of intra-particle diffusion model demonstrated that this model was not feasible to describe the adsorption process owing to a weak correlation. Moreover, Fig. 4b showed that the experimental data fitted the theoretic pseudo-second-order model simulated curves fairly well. These results indicated that chemical adsorption might be the rate-limiting step that controls the adsorption MB onto adsorbent, which were consistent with the previous studies reported in literatures^52^.

**Table 2:** Kinetic constants of MB on CA.

Kinetic models	Parameters	CA
Pseudo-first-kinetic models	*q*_e_ (mg/g)	103.94
	*K*_1_ (1/min)	0.02559
	*R*^2^	0.989
Pseudo-second-kinetic models	*K*_2_ (g/(mg min)	0.0002206
	*h* (mg/(g min))	40.44
	*R*^2^	0.996
Intra-particle diffusion equation	*K*_3_ (mg g^−1^ min^−1/2^)	5.9978
	*C*	24.2520
	*R*^2^	0.966

**Table 3:** Comparison of adsorption capacities of other biomass-derived adsorbents for removal of MB.

Adsorbent	Qm (mg/g)	Preparing temperature (°C)	Reference
Phosphoric acid treated corncob	140.25	160	This study
Acetic acid treated rice bran	25.1	90	^25^
Waste apricot-based activated carbon	102.04	500	^53^
Sulfuric acid treated Parthenium	88.29	300	^54^
Zinc chloride treated *Stipa tenacissima*	178.44	600	^55^
Sodium hydroxide treated corn stalk	49.01	75	^56^
Tartaric acid treated bagasse	57.14	120	^57^
Surfactant-modified pineapple leaf powder	52.6	90	^58^

### Comparison with other adsorbents

Comparisons of the maximum adsorption capacity (Qmax value) of CA with other biomass-derived adsorbents are presented in Table 3^25,53–58^. The CA shows the comparable adsorption capacity for MB compared to other biomass-derived adsorbents. And given its adsorption capacity and preparation at low temperature, CA was promising for MB removal from the waste water.

### Desorption studies

**Figure 7:**
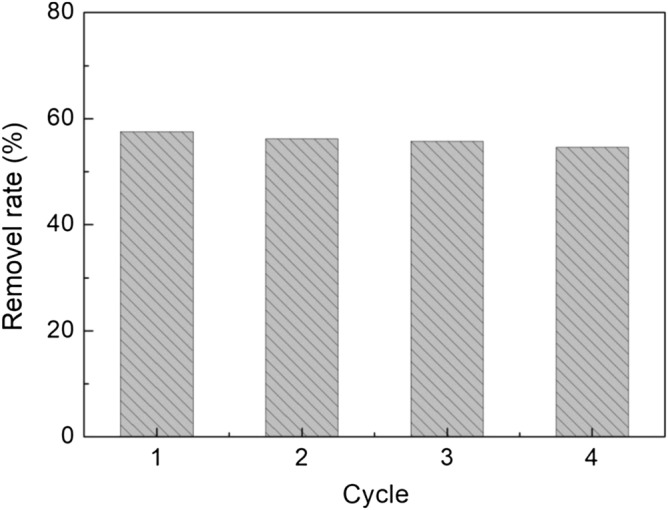
The removal rate of MB was employed to evaluate their operational stability.

The reusability of the adsorbent is also very important for its potential commercial application and economic feasibility in wastewater treatment. The tests of MB desorption were carried out with four times repeated batch adsorption–desorption cycles as shown in Fig. 7. It can be observed that CA still retained satisfactory adsorption efficiency of MB molecules even after four cycles of reuses, indicating the good reusability of CA.

## Conclusions

In this work, an effective modification method based corncob with phosphoric acid through HTC at low temperature conditions is described. As we know, this is the first report of the synthesis of carbonaceous adsorbent from lignocellulosic biomass by using a low-temperature hydrothermal method. A large number of oxygen-containing groups and pore channels were introduced, resulting in a significantly enhance in the as-obtained adsorbent adsorption capability. The synthesized carbonaceous adsorbent may be used for organic pollutants removal from water treatment at large scale economically.
